# Effectiveness of Antenatal Clinics to Deliver Intermittent Preventive Treatment and Insecticide Treated Nets for the Control of Malaria in Pregnancy in Mali: A Household Survey

**DOI:** 10.1371/journal.pone.0092102

**Published:** 2014-03-20

**Authors:** Jenny Hill, Kassoum Kayentao, Mahamoudou Touré, Sory Diarwara, Jane Bruce, James Smedley, Ogobara K. Doumbo, Feiko O. ter. Kuile, Jayne Webster

**Affiliations:** 1 Department of Clinical Sciences, Liverpool School of Tropical Medicine, Liverpool, United Kingdom; 2 Malaria Research and Training Centre, University of Sciences, Techniques and Technologies of Bamako, Bamako, Mali; 3 Disease Control Department, London School of Tropical Medicine and Hygiene, London, United Kingdom; Tulane University School of Public Health and Tropical Medicine, United States of America

## Abstract

**Background:**

WHO recommends intermittent-preventive-treatment (IPTp) with sulphadoxine-pyrimethamine (SP) and insecticide-treated-nets (ITNs) to prevent malaria in pregnancy in sub-Saharan Africa, however uptake remains unacceptably low. We evaluated the effectiveness of antenatal clinics (ANC) to deliver two doses of IPTp and ITNs to pregnant women in Segou district, Mali.

**Methods:**

We used household data to assess the systems effectiveness of ANC to deliver IPTp and ITNs to pregnant women and used logistic regression to identify predictors of ANC attendance, receipt of IPTp and ITN use during pregnancy, and the impact on community effectiveness.

**Results:**

Of 81% of recently pregnant women who made at least one ANC visit, 59% of these attended during the eligible gestational age for IPTp. Of these, 82% reported receiving one dose of SP and 91% attended ANC again, of whom 66% received a second dose, resulting in a cumulative effectiveness for 2-dose IPTp of 29%, most of whom used an ITN (90%). Cumulative effectiveness of 2-dose SP by directly observed therapy (DOT) was very low (6%). ITN use was 92%, and ANC was the main source (81%). Reported and ANC-card data showed some doses of SP are given to women in their first trimester. Women were less likely to receive two doses by DOT if they were married (OR 0.10; CI 0.03, 0.40), or lived <5 km from the health facility (OR 0.34; CI 0.14, 0.83). A high household person-LLIN ratio predicted low ITN use in pregnant women (OR 0.16; CI 0.04, 0.55).

**Conclusion:**

Our findings suggest poor adherence by health workers to provision of IPTp by eligible gestational age and DOT, contributing to low effectiveness of this strategy in this setting. ITN delivery and use among women was substantially higher. Efforts to improve health worker adherence to IPTp guidelines are needed to improve service delivery of IPTp.

## Background

Since 2004, the World Health Organization (WHO) has recommended a package of intermittent preventive treatment (IPTp) and insecticide treated nets (ITNs) together with effective management of clinical malaria and anaemia for malaria prevention and control during pregnancy in areas of stable malaria transmission in sub-Saharan Africa [1]. Until 2012, WHO recommended two doses of sulphadoxine-pyrimethamine, (SP) administered in the second and third trimesters of pregnancy [1]. These interventions can substantially reduce disease burden and adverse outcomes of malaria in pregnancy [2]–[4], and are inexpensive and cost effective [5]. Although these interventions have been available for over two decades [2]–[4], access to and use by pregnant women remains unacceptably low [6].

The Roll Back Malaria Partnership aims to ensure that by 2015, all pregnant women living in areas of high-intensity transmission in sub-Saharan Africa receive IPTp and use ITNs [7]. IPTp with SP is delivered to pregnant women through antenatal clinics (ANC) alongside ITNs. Despite almost universally high levels of ANC coverage in sub-Saharan Africa, coverage estimates for IPTp and ITNs in sub-Saharan Africa have increased only modestly between 2007 and 2010, from 13.6% to 21·5% and from 17.0% to 38·8% for IPTp and ITNs respectively, representing substantial missed opportunities at ANC [6]. According to the Countdown to 2015 Decade Report, in 20 countries with data, IPTp and ITNs, together with case management of malaria during pregnancy, have the lowest coverage among all the interventions delivered to pregnant women at ANC [8]. The reasons for this are diverse and stem from barriers to delivery as well as user behaviours, as described in a recent systematic review of the factors affecting the delivery, access and use of IPTp and ITNs [9]. For example two studies reported that health workers do not always offer IPTp to women at ANC [10], [11]. It is a particular concern that health services have been unable to deliver IPTp-SP effectively, as this relatively simple and cost effective regimen will most likely be replaced with more complicated and expensive drug regimens or new strategies [12] in the near future due to increasing resistance to SP [3], [13]. These alternative prevention strategies will present even greater challenges for effective delivery or systems effectiveness [14], [15].

A recent systematic review identified very few studies that evaluated the effectiveness of the delivery of IPTp [16] and ITNs [17], [18] through ANC. New methods are required to measure the relative impact of the different barriers to the delivery of these important interventions in order that programme managers can achieve the best value for money by prioritising which barriers to target in order to improve uptake. This study used household survey data to measure the effectiveness of the delivery of IPTp-SP and ITNs to pregnant women through ANC in one district in Mali, and assessed the predictors of effective delivery.

## Methods

### Ethical Considerations

The study was approved by the ethical committees of the Faculty of Medicine, Pharmacy and Odonto-stomatology of Bamako, the Liverpool School of Tropical Medicine, and the London School of Hygiene and Tropical Medicine. Written informed consent was obtained from women prior to being interviewed at home and for adolescents who were not considered mature minors (a person under 18 years of age who is pregnant, married or a parent), written informed consent was obtained from the head of the household.

### Study Area

The household survey was conducted as part of a larger study to identify and quantify the major barriers to the scale up and use of interventions to control malaria in pregnancy at the district, facility, and community levels in Mali. The study was conducted in Segou District, Segou Region. Malaria in Segou Region is seasonal ranging from holo-endemic in the southern part of the district and meso-endemic to the north. HIV prevalence is 1.3% in the general population of Segou and 1.7% in women aged 15–49 years [19]. Segou District has a total population of 448,552 projected from the 1998 census, with more than 60% of this population living in rural areas. The most common ethnic groups are Bamanan and Sarakole/Soninke, and the main economic occupation of the occupants is subsistence agriculture.

The health care system in Mali is severely under-resourced. The government funds health facilities down to district level only, comprising one hospital and one district level health centre termed the Centre de Santé de Reference (CSRef). All health facilities below this level, at what is termed the operational level, are funded by communities themselves through a concept developed initially as the ‘Bamako Initiative’ [20]. This engagement was done to push external partners and the government to transfer responsibility to the community for the creation, organisation and management of community health services (CSCom) through the ASACO (community health association) created in each ‘health area’. At the time of the study, Segou District had a total of 26 functioning health structures, 18 of which were owned by the community (i.e. headed by a nurse paid by the community) and 8 of which were headed by a physician paid by the government. The government facilities comprised 1 hospital and 1 district level health facility (CSRef) based in Segou town (district headquarters).

Malaria in pregnancy services provided through Consultation Prénatale (CPN, or ANC) include 2 doses of IPTp administered by directly observed therapy (DOT) between month 4 and 8 (inclusive) gestation, with each dose given at least one month apart, and 3 doses for women who are HIV positive, and provision of a free long-lasting insecticide treated net (LLIN) to all women at first ANC visit in areas of high transmission [21].

National level Demographic and Health Survey (DHS) data available at the time the study was designed (DHS 2006) showed that 63% of women made two or more ANC visits, 4% pregnant women received two doses of IPTp-SP during ANC visits and 39% of pregnant women used an ITN the night before the survey [19]. The IPTp and ITN coverage figures for Segou Region were similar to the national average, with 3.8% of pregnant women reporting having received two doses of IPTp and 43% reporting having used an ITN the night before the survey.

### Study Design and Selection of Study Participants

A two stage cluster sampling household survey was undertaken over a period of 3 weeks in September 2009 during the rainy season. Enumeration areas from the 1998 census were used to select 40 villages, representing clusters, with probability proportional to size. The primary endpoint for sample size calculation was the proportion receiving 2-doses of IPTp. A sample size of 213 women of child-bearing age (15–49 years) was sufficient to estimate an uptake of 50%, with a precision of 6%, a design effect of 1.75 and 10% non-response. To obtain an evaluable sample of 213 women, the initial sample size was set at 624 [22] based on the hypothetical assumptions that 53% of women attending ANC are offered the first dose of IPTp, of which 99% take it, of which 65% return for a second ANC visit, of which 100% receive the second dose of IPTp (624×0.53×0.99×0.65 = 213).

A total of 16 households were randomly selected within each cluster using a modified EPI sampling technique. By using this method the sample was self weighted. One respondent within each household was selected using a set of predefined criteria as follows: women must be aged 15–49 years; pregnant women were prioritised over mothers of children aged under one year, who were prioritised over other non-pregnant women; and if more than one woman was currently pregnant/mother of a child aged under one year/aged 15–49 years, the woman most closely related to the head of the household was interviewed (i.e. wife> daughter> sister> niece/aunt> daughter-in-law).

### Data Collection Instrument

The questionnaire sought to obtain key coverage indicators on the frequency and timing of ANC attendance, the frequency, timing and source of IPTp-SP doses with or without DOT, ITN use during the current/most recent pregnancy and source. Additional questions explored women’s knowledge of malaria in pregnancy in relation to use of IPTp and ITNs, and whether HIV status alters behaviour patterns and uptake. A composite malaria knowledge score was created based on the cumulative score of correct responses in relation to source of malaria (mosquitoes), consequences of malaria in pregnancy to mother (anaemia) and unborn child (miscarriage, low birth weight (LBW), premature birth, still birth) and of methods to prevent malaria in pregnancy (e.g. ITNs, nets and/or ITNs). HIV status was self-reported, and additional indirect questions about recognition and use of cotrimoxazole and history of episodes of opportunistic infections associated with HIV were included. Self-reported IPTp, ITN and ANC practices, including HIV testing and results, were compared with data contained in ANC cards where available. Recording of household assets to develop a wealth index and observation of household ITNs *in situ* was also performed. Principle components analysis was used to construct a wealth index in order to assess the effect of socio-economic status (SES) [23], [24]. Household characteristics such as education of the household head, floor material, source of drinking water, type of toilet facility, household ownership, cooking fuel type, and ownership of a television and watch were used in the Principle components analysis.

The questionnaire was translated and back translated to ensure accuracy of translation of concepts and variables, and then pre-tested. Interviewers were trained to conduct the interviews in the national spoken language of Bambara, translated directly from the French translation of the questionnaire.

### Data Processing and Analysis

Data from the household surveys were collected on personal digital assistants. All analyses were adjusted for the survey design and clustering of households using STATA 11.

This analysis was restricted to pregnant women and mothers of children under one year, termed ‘recently pregnant women’, given likely use and potential recall of IPTp and ITNs. Socio-demographic characteristics of currently and recently pregnant women were described and compared. Proportions of women accessing ANC, receipt and timing of each dose of IPTp-SP from any source and receiving ITNs from any source were quantified. The indicator for ITN use in currently pregnant women was the proportion of pregnant women who reported having used an ITN the night before the survey [25], [26], and for recently pregnant women, the proportion of women who reported having used an ITN regularly during their most recent pregnancy [27], [28].

We used a framework developed previously for effectiveness analysis, adapted specifically for the delivery of IPTp-SP and ITNs through ANC to measure delivery system effectiveness using household data to identify critical points in the delivery system which were ineffective [9]. The systems effectiveness analysis was undertaken using women who had been recently pregnant. We excluded HIV positive women with documented use of cotrimoxazole, since IPTp with SP is not recommended for women taking cotrimoxazole due to potential severe adverse events caused by concomitant use of two sulphur-containing drugs [1]. Systems effectiveness was defined as the proportion of women attending ANC who reported having received a full course of IPTp-SP (two doses of SP) and having used an ITN regularly during the most recent pregnancy. Two categories of effectiveness analyses were applied - ‘intermediate process effectiveness’ and ‘cumulative systems effectiveness’ [29]. Intermediate process effectiveness represents coverage of women at each of the intermediate steps in the delivery of IPTp-SP, with the addition of ‘used an ITN’ and then ‘used an ITN from ANC’ together with taking the second dose of IPTp as the final process. Intermediate process effectiveness was calculated as the number of women who successfully completed the intermediate process (the numerator) as a proportion of all women who reached that point in the delivery system (the denominator). An intermediate process was described as ineffective if ≤80% women successfully completed that step. Cumulative systems effectiveness represents successful coverage of women for each of the intermediate steps up to that point in the delivery system. The final step represents the percent of eligible women in the community that received both interventions and is equivalent to a cumulative coverage indicator for several indicators (two ANC visits, two doses of SP (IPTp) and ITN use) that are measured by DHS.

The effectiveness analyses for IPTp were performed on women first attending ANC in an eligible month of gestation (4 to 8 months) for IPTp and excluded women who were taking cotrimoxazole as documented on ANC cards. We did not take into account the dosing interval. These analyses were conducted under two scenarios, Scenario A where the criteria of receiving IPTp-SP by DOT was not included, and Scenario B where receiving IPTp-SP by DOT was included. The systems effectiveness analysis for ITNs was not restricted by gestational age as policy states ITNs are to be delivered to women at their first ANC visit regardless of gestational age.

Univariate analyses of potential predictors of receipt of: i) at least one ANC visit between 4–8 months gestation ii) two doses of IPTp-SP (with or without DOT); iii) one dose of SP by DOT; and iv) two doses of SP by DOT (as the four least effective processes identified), were conducted. Logistic regression models for survey design were used, and adjusted Wald tests were applied to test for associations between predictors and outcomes. Predictors of ITN use explored included selected individual and household socio-demographic factors (women’s age, marital status, education, malaria knowledge using the composite malaria knowledge score, residence, and SES); person: net ratio stratified according to any net, ITNs, and LLIN; gravidity; losing a live born child; number and timing of ANC visits; whether they recently had a malaria episode and whether they took medicine for that episode. Potential predictors significant at the 10% level (p≤0.1) in the univariate analyses were included in multivariable logistic models to determine which factors were associated with the outcomes after accounting for other potential predictors. Interactions between paired predictors were assessed for all predictors included in the models.

We estimated the potential and actual effectiveness of the IPTp strategy on LBW extrapolated to the pregnant population of Segou Region (n = 116,813). We used the protective efficacy of IPTp on LBW of 35% among women protected by 2-doses of IPTp-SP and an ITN in any pregnancy observed in a cross sectional study from 2007 to 2009 in 5 districts of Mali (adjusted prevalence ratio (APR) 0.57; 95% CI 0.40, 0.80) and an incidence of LBW of 12% in women not receiving IPTp (Kassoum Kayentao, personal communication), and co-coverage with IPTp by DOT and ITNs, using the following formula [30]:
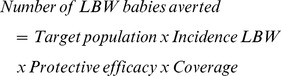



## Results

### Study Population

A total of 637 women of childbearing age were interviewed (99.5% response rate) of which 150 were pregnant (24%) and 264 (41%) were recently pregnant women. Among the pregnant women, the majority were in their second or third trimester (87%, 132/150) and the mean gestational age was 6 months (IQR 4–8) (Table 1). The majority of women were multigravidae, with a median number of births in the pregnant and recently pregnant groups of 3 (IQR 2–6) and 4 (IQR 2–6) respectively. The median age was 27 years (IQR 22–30) among pregnant women and 25 years (IQR 21–30) among recently pregnant women. The majority of women in both groups were married (>90%), and lived in rural areas (>67%). The main ethnic group was Bambara (>55%) and about two-thirds of women had no education (>62%). Almost half (45%; 60/134, 16 missing values) of pregnant women and over a third of recently pregnant women (37%; 99/265) had lost a live born child. Self reported HIV prevalence was very low, 0% among pregnant women and 2% (1/50) among recently pregnant women.

**Table 1:** Socio-demographic characteristics of the respondents.

Socio-demographic data	Pregnant women (N = 150)		Recently pregnant women (N = 264)	
Characteristic	n	% (95% CI)	n	% (95% CI)
**Trimester**				
1^st^ trimester	19	12.7		
2^nd^ trimester	62	41.3		
3^rd^ trimester	70	46.0		
Median months gestational age	150	6 (IQR 4–8)		
**Gravidity**				
Gravida 1	16	10.7	47	18.4
Gravida 2	20	13.3	48	18.8
Gravida 3>	114	76.0	160	62.8
Median number of births	150	3 (IQR 2–6)	264	4 (IQR 2–6)
**Age group (15–49 years)**				
15to19	23	15.3	46	17.4
20to24	27	18.0	61	23.1
25to29	47	31.3	64	24.2
30to34	34	22.7	56	21.2
35to39	11	7.3	24	9.1
40+	8	5.3	13	4.9
Median Age		27 (IQR 22–30)		25 (IQR 21–30)
**Marital status**				
Married	145	96.7	245	92.8
Divorced/separated	1	0.7	0	0.0
Single	4	2.7	19	7.2
**Location of residence**				
Urban	45	30.0	6	32.6
Rural	105	70.0	178	67.4
**Education level**				
None/nursery	100	66.7	164	62.1
Primary	46	30.7	83	31.4
Secondary/College/University	4	2.7	17	6.4
**Ethnic Group**				
Bambara	87	58.0	146	55.3
Peulh	16	10.7	38	14.4
Malinke	4	2.7	8	3.0
Bozo/Somono	4	2.7	5	1.9
Dogon	1	0.7	4	1.5
Other	38	25.3	63	23.9
**HIV status (self reported)**	0/32	0	1/50	2.0 (0.3, 14.2)
**Women with child aged <1 year**	5/134*	3.7 (1.6, 8.5)	264/264	100.0 (−)
**Women with child aged <5 years**	110/134*	82.1(74.0,88.1)	264/264	100.0 (−)
**Woman has lost a child**	60/134*	44.8 (36.1,53.8)	99/264	37.5 (30.5,45.1)

N, denominator; n, numerator; CI, confidence interval; IQR, inter-quartile range; HIV, human immunodeficiency virus.

*excludes primigravid women.

### Coverage Indicators

#### Access to ANC and timing of first ANC visit

**Figure 1:**
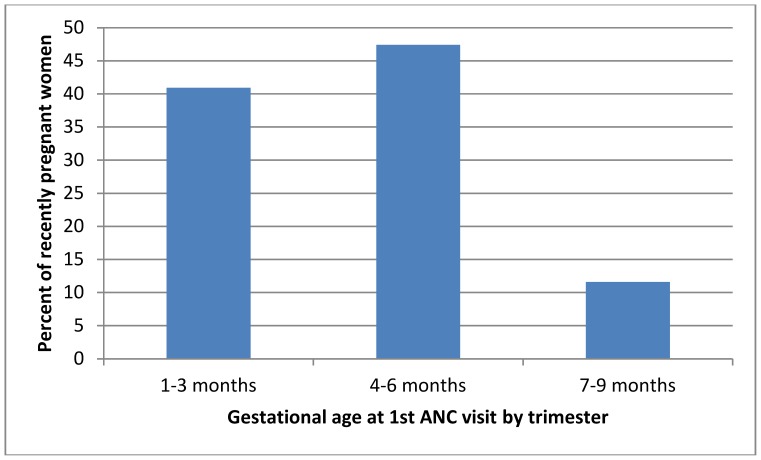
Timing of first ANC visit among recently pregnant women who attended ANC (N = 215). ANC, antenatal clinic.

Among recently pregnant women, 81% (215/264) of women reported attending ANC at least once, 75% (198/264) at least twice and 42% (112/264) made four or more visits, with a median number of 4 ANC visits (IQR 3–5) (Table 2). Just over 40% of women first attended ANC in their first trimester, with almost half initiating attendance in their second trimester (47%) (Figure 1). Attendance among pregnant women was lower (63% for one visit and 43% for two visits), with a median of 2 visits (IQR 1–3), as would be anticipated given that 54% of women were still in their first or second trimester (Table 2). The median month of gestation at first ANC visit was 4 months in both groups of women (IQR 3–5).

**Table 2:** Key coverage indicators for ANC attendance and malaria in pregnancy interventions among pregnant and recently pregnant women.

Coverage Indicators		Pregnant women (N = 150)		Recently pregnant women (N = 264)	
		n/N	% (95% CI)	n/N	% (95% CI)
**Number ANC visits**					
At least one visit		94/150	62.7 (52.8, 71.6)	215/264	81.4 (75.2,86.4)
At least two visits		64/150	42.7 (33.6, 52.2)	198/264	75.0 (68.2, 80.8)
4 or more visits		23/150	15.3 (9.7, 23.4)	112/264	42.4 (34.6, 50.6)
Median number of ANC visits among ANC attendees		94	2 (IQR 1–3)	215	4 (IQR 3–5)
**Gestational age at 1^st^ ANC visit**					
0–3 months		34/93	36.6 (25.6,49.1)	88/215	40.9 (33.0,49.3)
4–6 months		53/93	57.0 (45.8,67.5)	102/215	47.4 (40.4,54.6)
7–9 months		6/93	6.5 (2.7,14.5)	25/215	11.6 (7.2,18.2)
Median gestational age at 1^st^ ANC visit		92	4 (IQR 3–5)	208	4 (IQR 3–5)
**1 dose SP** †					
1 dose (all trimesters, any source)				199/264	75.1 (68.3, 80.8)
1 dose (4–8 months, any source)				150/264	56.6 (50.1, 62.9)
Dose 1 at ANC (all trimesters)				177/199	88.9 (83.3, 92.8)
Dose 1 by DOT at ANC (all trimesters)				60/173≠	33.9 (23.6, 46.0)
**2 doses SP** †					
2 doses (all trimesters, any source)				115/262	43.4 (36.5, 50.5)
2 doses (4–8 months, any source)				103/262	38.9 (32.5, 45.7)
Dose 2 at ANC (all trimesters)				110/115	96.7 (90.0, 98.2)
Dose 2 by DOT at ANC (all trimesters)				28/108≠	25.5 (17.2, 36.0)
**3 doses SP** †					
3 or more doses (all trimesters, any source)				21/260	8.1 (4.8, 13.2)
**ITN use (amongst sampled women)**					
ITN used last night		138/149	92.6 (84.7, 96.6)		
ITN used during last pregnancy				242/263	92.0 (83.0, 94.8)
ITN sourced from ANC clinic		102/138	73.9 (65.8, 80.7)	196/242	81.0 (75.4, 85.6)
**Co-coverage IPTp** † **and ITNs**					
**1 dose SP**	No IPTp1, No ITN			10/262	3.8 (2.0, 7.1)
	IPTp1, No ITN			11/262	4.2 (2.2, 7.7)
	No IPTp1, ITN			53/262	20.2 (15.1, 26.6)
	Both IPTp1 & ITN			188/262	71.5 (65.2,77.0)
**2 doses SP**	No IPTp2, No ITN			14/260	5.3 (3.1, 8.9)
	IPTp2, No ITN			7/260	2.7 (1.2, 5.9)
	No IPTp2, ITN			131/260	50.4 (43.0, 57.8)
	Both IPTp2& ITN			108/260	41.5 (35.2, 48.2)

IPTp1, one dose of SP; IPTp2, two doses of SP; DOT, directly observed therapy; ITN, insecticide treated net; ANC: antenatal clinic; IPTp, intermittent preventive treatment; N, denominator; n, numerator; CI, confidence interval; IQR, inter-quartile range; SP, sulfadoxine-pyrimethamine.

† Excludes women who did not receive IPTp and who were taking cotrimoxazole as documented on ANC cards.

≠ missing records.

#### Number of IPTp doses and timing of receipt

Three-quarters of recently pregnant women (not taking cotrimoxazole) reported receiving one dose of SP in any trimester from any source (75%; 199/264), and 57% (150/264) in women who received doses in an eligible month (Table 2). The majority of women reported receiving the first dose of SP at ANC (89%; 177/199) but only 34% said they received it under DOT (60/173, 4 missing values). Only 43% (115/262) of recently pregnant women reported receiving the recommended second dose of SP (any trimester, any source), and only 39% (103/262) in an eligible trimester. The majority (97%) of women reported receiving the second dose of SP from ANC of which only a quarter (26%) reported receiving it by DOT. A small proportion of women (8%) reported receiving three doses of SP in any trimester from any source.

#### IPTp coverage by number and timing of ANC visits

**Figure 2:**
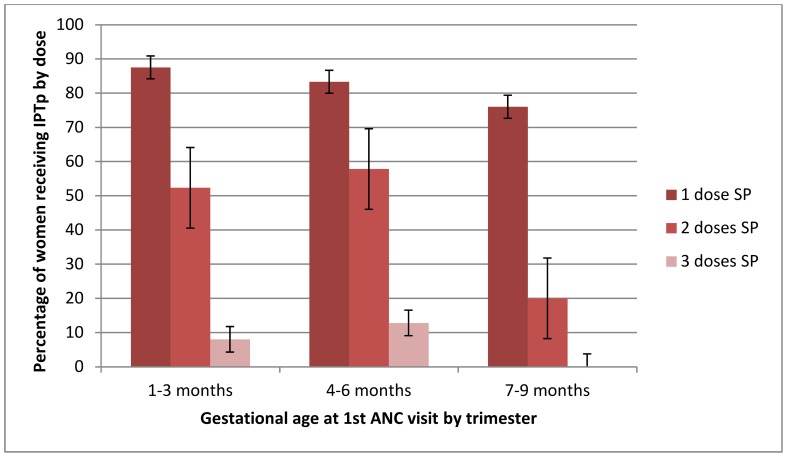
Number of IPTp doses received by trimester at 1^st^ ANC visit among recently pregnant women. IPTp, intermittent preventive treatment; ANC, antenatal clinic.

Among recently pregnant women the mean number of doses of SP showed an increasing trend with increasing number of ANC visits. Similarly, the number of doses of SP received was associated with gestational age at first ANC visit, with fewer recently pregnant women receiving a second dose of SP if they first attended ANC in their second or third trimesters (p = 0.01) (Figure 2).

#### ITN use and co-coverage with ITNs and IPTp

Among pregnant women, 93% (138/149) reported using an ITN the previous night and similarly 92% (242/263) of recently pregnant women reported using an ITN regularly during their most recent pregnancy. Approximately three-quarters of women in both groups reported obtaining the net used in pregnancy from ANC (74% and 81% of pregnant and recently pregnant women respectively). In terms of co-coverage of ITN use and IPTp receipt among recently pregnant women, 72% (188/262) reported using an ITN and receiving one dose of SP and 42% (108/260) of using an ITN and receiving two doses of SP. Whilst few women reported using neither ITN nor one or two doses of SP (4% and 5% respectively), a large proportion of women reported using an ITN alone in the absence of any SP, with 20% (53/262) and 50% (131/260) of ITN users who reported not having received one and two doses of SP respectively.

### System Effectiveness of IPTp and ITN Delivery through ANC

#### IPTp with or without DOT

**Figure 3:**
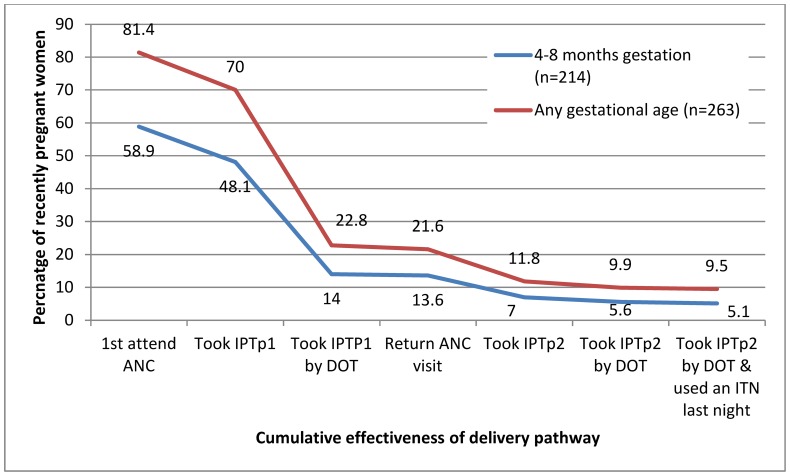
Cumulative effectiveness† of ANC to deliver IPTp and ITNs among recently pregnant women. IPTp, intermittent preventive treatment; IPTp1, first dose; IPTp2, second dose; ANC, antenatal clinic; DOT, directly observed therapy; ITN, insecticide treated net. †Cumulative systems effectiveness represents successful coverage of women for each of the intermediate steps up to that point in the delivery system.

Although 81% of recently pregnant women visited ANC at least once (Table 1), only 59% (125/213) of women not taking co-trimoxazole made their first ANC visit in an eligible gestational age for receiving IPTp (Figure 3). Of these, 82% (102/125) received the first dose of SP, and of the 91% (93/102) of women who returned for a second ANC visit, only 66% (61/92) received a second dose (Table 3). Only 24% (55/213) of women received the recommended two doses of SP and used an ITN during pregnancy. There were two intermediate processes in the delivery system that were ≤80% effective – women first attending ANC in an eligible gestational age for IPTp, and the receipt of the second dose of SP. ITN use among women receiving two doses of SP was >90%.

**Table 3:** Intermediate process and cumulative effectiveness± of receiving two doses of SP and using an ITN with and without DOT in an eligible gestational age (4–8 months) among recently pregnant women not taking co-trimoxazole.

	2 doses of SP				2 doses of SP by DOT			
Intermediate process	N	n	Intermediate process effectiveness± % (95% CI)	Cumulative deliveryeffectiveness≠ %	N	n	Intermediate processeffectiveness± % (95% CI)	Cumulative deliveryeffectiveness≠ %
**Attend ANC at least once**	213	125	58.7 (50.3, 66.6)	58.7	213	125	58.7 (50.3, 66.6)	58.7
**Took IPTp1**	125	102	81.6 (72.1, 88.4)	47.9	125	102	81.6 (72.1, 88.4)	47.9
**Took IPTp1 by DOT**					91†	30	33.0 (19.7, 49.7)	15.8
**Attend ANC at least twice**	102	93	91.2 (82.2, 95.8)	43.7	30	29	96.7 (77.4, 99.6)	15.3
**Took IPTp2**	92	61	66.3 (57.4, 74.2)	29.0	29	15	51.7 (34.3, 68.7)	7.9
**Took IPTp2 by DOT**					15	12	80.0 (49.9, 95.1)	6.3
**Took IPTp2 & used an ITN** **during last pregnancy**	61	55	90.2 (78.2, 95.9)	26.2				
**Took IPTp2 & used an ** ***ANC*** ** ITN** **during last pregnancy**	55	51	92.7 (81.6, 97.3)	24.3				
**Took IPTp2 by DOT & used an ** ***ANC*** **ITN during last pregnancy**					12	11	91.7 (46.8, 99.3)	5.8

IPTp1: one dose of SP; IPTp2: two doses of SP; DOT: directly observed therapy; ITN: insecticide treated net; ANC: antenatal clinic; IPTp, intermittent preventive treatment; SP, sulfadoxine-pyrimethamine; N, denominator; n, numerator; CI confidence interval.

† 11 missing records.

± Intermediate process effectiveness represents coverage of women at each of the intermediate steps in the delivery of IPTp;

≠ Cumulative systems effectiveness represents successful coverage of women for each of the intermediate steps up to that point in the delivery system.

#### IPTp given by DOT

With respect to DOT, the cumulative system effectiveness was substantially lower, with only 6% of women receiving two doses of SP by DOT and using an ITN (Table 3) (Figure 3). The intermediate processes that were ≤80% effective were, in addition to first attending ANC in an eligible gestational age for IPTp, receipt of the first dose of SP under DOT (33%), receipt of a second dose of SP (52%) and receipt by DOT (80%). ITN use among women receiving two doses of SP by DOT was 92%.

#### ITNs

Among the 81% of recently pregnant women who attended ANC at least once, 93% (200/215) reported using an ITN during their last pregnancy of which 85% (169/200) were obtained from ANC (Table 4). Among the 63% (94/150) of pregnant women who attended ANC at least once, 95% (88/93) reported using an ITN the previous night, of which 86% (76/88) were sourced from ANC.

**Table 4:** Intermediate process and cumulative effectiveness± of receiving an ITN from ANC among pregnant (all gestational ages) and recently pregnant women.

	Pregnant women				Recently pregnant women			
Intermediate process	N	n	Intermediate processeffectiveness± %(95% CI)	Cumulative deliveryeffectiveness^≠^ %	N	n	Intermediate processeffectiveness± %(95% CI)	Cumulative deliveryeffectiveness^≠^ %
**1. Attend ANC at least once**	150	94	62.7 (52.8, 71.6)	62.7	264	215	81.4 (75.2, 86.4)	81.4
**2. Used an ITN last night/during** **last pregnancy**	93	88	94.6 (87.5, 97,8)	58.7	215	200	93.0 (88.6, 95.8)	75.8
**3. Used an ** ***ANC*** ** ITN last night/during** **last pregnancy**	88	76	86.4 (76.2, 92.6)	50.7	200	169	84.5 (78.1, 89.3)	64.0
**Difference in coverage between** **process 1 and 3**				12.0				17.4

N, denominator; n, numerator; CI, confidence interval; ITN: insecticide treated net; ANC: antenatal clinic.

±Intermediate process effectiveness represents coverage of women at each of the intermediate steps in the delivery of IPTp; ^≠^Cumulative systems effectiveness represents successful coverage of women for each of the intermediate steps up to that point in the delivery system.

### Predictors of IPTp Receipt

#### Two doses of SP

The univariate predictors of recently pregnant women reporting having received two doses of SP were: number of ANC visits and timing of 1^st^ ANC visit (Table 5). The odds of women reporting receipt of two doses of SP progressively decreased the later the woman made her first ANC visit (OR 0.24, 95% CI 0.09, 0.67 for 7–9 months vs. 1–3 months gestation) and increased with number of ANC visits (OR 12.00, 95% CI 1.71, 47.91 for 3 vs. 1 visit). After adjustment, no factors were independently associated with receipt of two doses of SP among recently pregnant women (Table 5).

**Table 5:** Univariate and multivariate analysis of predictors of receiving *two doses of IPTp* among recently pregnant women.

	Recently pregnant women (N = 263)							
Determinant	n	%	OR	95% CI	P value	AOR	95% CI	P value
**Number of ANC visits**					0.02			0.1
1 visit	16	12.5	1			1		
2 visits	28	35.7	3.89	0.67,22.45		2.90	0.48,17.71	
3 visits	57	63.2	12.00	2.24,64.40		8.00	1.33,48.30	
4> visits	110	56.4	9.04	1.71,47.91		6.14	1.01,37.41	
**Timing of 1st ANC visit by trimester**					0.01			
0–3 months	86	53.5	1			1		0.1
4–6 months	102	57.8	1.19	0.66,2.15		1.38	0.75, 2.55	
7–9 months	23	21.7	0.24	0.09,0.67		0.47	0.15, 1.45	
**Malaria knowledge score**					0.06			0.3
Score 1 (Lowest)	75	32.0	1			1		
Score 2	94	48.9	2.04	1.09, 3.82		1.90	0.85, 4.28	
Score 3 (Highest)	92	48.9	2.03	1.00,4.16		1.61	0.65,3.98	

N, denominator; n, numerator, OR, odds ratio; AOR, adjusted odds ration; CI, confidence interval ANC, antenatal clinic.

#### One and two doses of SP by DOT

The univariate predictors of recently pregnant women reporting that they received one dose of SP by DOT were SES and marital status, and for two doses of SP by DOT, marital status and distance to the local health facility (Table 6). After adjustment, being unmarried was an independent predictor for receiving either dose of SP by DOT (AOR 0.10, 95% CI 0.03, 0.40 for married women receiving 2 doses by DOT), and women who lived >5 km from the local health facility were also more likely to report receipt of two doses of SP by DOT (AOR 0.34, 95% CI 0.14, 0.83 for women living <5 km from a health facility).

**Table 6:** Univariate and multivariate analysis of predictors of receiving one and two doses of SP *under DOT* among recently pregnant women.

Determinant	1 dose SP under DOT (N = 174)								2 doses SP under DOT (N = 109)							
	n	%	OR	95% CI	P value	AOR	95% CI	P value	n	%	OR	95% CI	P value	AOR	95% CI	P value
**Married**					0.01			0.03					0.005			0.002
No	11	72.7	1			1			7	71.4	1			1		
Yes	163	31.9	0.18	0.04,0.70		0.19	0.04,0.85		102	22.6	0.12	0.03, 0.49		0.10	0.03,0.40	
**SES**					0.095			0.1								
1 most poor	28	14.3	1			1										
2	33	30.3	2.61	0.71,9.55		2.19	0.59,8.11									
3	41	46.3	5.18	1.47,18.24		4.82	1.37,16.99									
4	38	42.1	4.36	1.37,13.85		3.85	1.17,12.71									
5 least poor	33	33.3	3.00	0.85,10.58		2.54	0.69,9.37									
**Distance to facility**													0.03			0.02
≥5 km									33	39.4	1			1		
<5 km									76	19.7	0.38	0.16,0.90		0.34	0.14,0.83	

N, denominator; n, numerator, OR, odds ratio; AOR, adjusted odds ratio; CI, confidence interval ANC, antenatal clinic; DOT, direct observed therapy; SP, sulfadoxine-pyrimethamine; SES, socio-economic status; km, kilometres.

### Predictors of ITN Use

The univariate predictors of recently pregnant women reporting using an ITN in pregnancy were being married (OR 3.5, 95% CI 1.00, 12.60), age, distance form health facility and person: LLIN ratio. Adolescents were less likely to use ITNs than older women (OR 0.30, 95% CI 0.13, 0.74) as were women who lived <5 km from a health facility (OR 0.32, 95% CI 0.10, 1.04) and who lived in a household with a high person: LLIN ratio (OR 0.26 95% CI 0.06, 1.12 for 4+ vs. <2 persons per net) (Table 7). After adjustment the only independent predictor for using an ITN among recently pregnant women was person: LLIN ratio (AOR 0.16 95% CI 0.04, 0.55 for 4+ household members per LLIN.

**Table 7:** Univariate and multivariate analysis of predictors of *using an ITN* among recently pregnant women.

	Recently pregnant women (N = 263)							
Determinant	n	%	OR	95% CI	P value	AOR	95% CI	P value
**Parity**					0.06			0.4
Para 1	9	77.8	1			1		
Para 2–4	123	89.4	2.45	0.98,6.14		1.70	0.48,6.01	
Para 5>	131	95.4						
**Married**					0.05			0.7
No	19	79.0	1			1		
Yes	243	93.0	3.5	1.00,12.60		1.46	0.26,8.06	
**Adolescent**					0.01			0.2
No	216	94.0	1			1		
Yes	46	82.6	0.30	0.13,0.74		0.43	0.10,1.84	
**Person: LLIN ratio**					0.02			0.004
<2	44	95.5	1			1		
2–<4	101	96.0	1.15	0.21,6.25		0.82	0.18,3.65	
4+	58	84.5	0.26	0.06,1.12		0.16	0.04,0.55	
**Took medicine for malaria**					0.09			
No	15	80.0	1			NA		
Yes	133	92.5	3.08	0.82,11.56				
**Distance to facility**					0.06			0.09
≥5 km	86	96.5	1			1		
<5 km	176	89.8	0.32	0.10,1.04		0.23	0.04,1.29	

N, denominator; n, numerator, OR, odds ratio; AOR, adjusted odds ratio; CI, confidence interval; LLIN, long lasting insecticide treated net; km, kilometres; NA, Not available due to non convergence.

### Modelled Number of LBW Averted

The potential effectiveness of the malaria in pregnancy prevention strategy in averting LBW among the pregnant population of Segou Region was calculated using the effectiveness data from recently pregnant women not taking cotrimoxazole. This showed that 59% (126/214) made their first visit in an eligible trimester and 90% returned to ANC for a second visit (93/103) (Table 3) and could thus be potential recipients of two doses of SP by DOT and use of an ITN; and result in a potential 2,914 cases of LBW averted. However, based on the reported co-coverage of two doses of SP by DOT and ITNs (5%; 11/214), actual effectiveness was only 252 cases of LBW averted. The difference between potential and actual effectiveness is 2,662 cases of LBW (95% CI 152–4,335) that may have been averted if all women in Segou Region who attended ANC at least twice had received IPTp by DOT and used an ITN, equivalent to 228 (95% CI 13–371) cases of LBW per 10,000 women.

## Discussion

We have used household data to assess the ANC attendance patterns among pregnant women and the relative effectiveness of the health system in delivering IPTp and ITNs to women through ANC in Segou District in Mali. Whilst a high percentage of women made at least two ANC visits (75%), with a significant proportion of women initiating ANC attendance in their first or second trimester (88%), only a quarter of women received two doses of SP and used an ITN, and only 5% received both doses of SP by DOT and used an ITN. Both ANC attendance patterns among women and factors affecting the delivery of care at ANC contribute to the low effectiveness reported.

Women in this setting start their ANC visits earlier than reported in East Africa [31], and return for multiple visits, suggesting a favourable pattern of behaviour for the delivery of ANC services. ANC attendance in the study was slightly higher than reported in the most recent Mali DHS [19], with 75% compared to 63% of women making two or more ANC visits, a higher median of ANC visits (median of 4 compared to 3), and a higher proportion of women initiating ANC in their first trimester (41% compared to 30%, with a median of 4 months gestation). Therefore a high proportion of women were not eligible for the first dose of IPTp at their first ANC visit, which should only be given at the start of the second trimester.

Among the women who first attended ANC between 4–8 months gestation, a high proportion reported receiving one dose of SP, but only a third of doses were given by DOT. However, the majority of women who received a second dose of SP reported to have received it by DOT. The poor adherence to DOT, and the reasons for this, are described in a parallel study using observations and in-depth interviewers with health providers in a sample of health facilities in Segou District [32], [33]. Observation data collected in health facilities found provision of IPTp-SP by DOT to be very low, 0% and 34.3% (95% CI 10.5, 69.8) at district and community level health facilities, respectively. The main reason for poor adherence to giving IPTp by DOT reported by the majority of health workers interviewed was that they had decided, through personal experience, reports of pregnant women, training, or supervision that the side effects of SP were such that it should not be given to pregnant women on an empty stomach. It is possible that because the second dose of SP is given in the late second or third trimester, i.e. at a more advanced stage of pregnancy, that side effects and especially nausea, would be less prevalent hence the higher number of women who reported receiving the second dose by DOT. The incidence of side effects to SP by gestational age together with the perception of this by health workers requires further investigation. We undertook a similar study in Kenya where we found the least effective steps in the delivery of SP was receiving any SP, but when women did receive SP they were more likely to receive it by DOT than in Mali [9]. This type of analysis can therefore by useful for highlighting these programmatic deficiencies, which vary from country to country and also within the same country.

**Figure 4:**
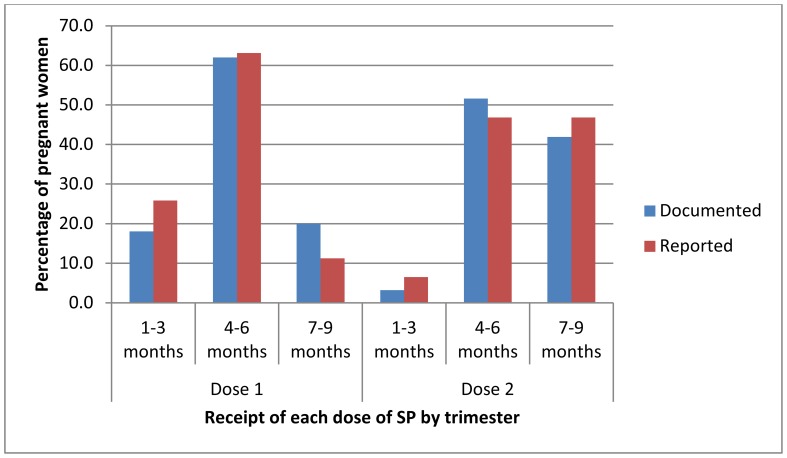
Documented and reported receipt of first and second dose of SP for IPTp by trimester among pregnant and recently pregnant women. IPTp, intermittent preventive treatment; SP, sulphadoxine-pyrimethamine.

Of concern is the finding that just over 20% of recently pregnant women reported receiving the first dose of SP in their first trimester, i.e. in an ineligible gestational age of pregnancy. This may to some extent reflect poor recall or knowledge among women, nevertheless ANC card data indicated a similar proportion of women were given IPTp in the first trimester, confirming that there was some practice of providers giving women the first dose of SP in their first trimester (Figure 4). Confusion among health providers over the timing of giving IPTp-SP has been described elsewhere [34], and in the parallel study in Mali, some health providers stated that women of less than one month gestation, rather than 1^st^ trimester, should not be given SP [32]. On the basis of a recent meta-analysis showing that three or more doses of SP are more effective than two doses [35], WHO recently updated its policy guidance to recommend the administration of SP to pregnant women at every scheduled antenatal visit starting in the second trimester [36]. The new guidance is more closely aligned with the new Focused Antenatal Care schedule of four visits in the second and third trimesters [36], and is anticipated to simplify the strategy and improve uptake of IPTp-SP. The release of this simplified guidance provides an opportunity for countries to improve the delivery of IPTp-SP, and to retrain health providers to avoid administering SP in the first trimester.

As anticipated by the high ANC coverage data in Segou District, a high proportion of women returned to ANC for a further visit in an eligible gestational age after receiving the first dose of SP (>90%). However, only just over half of these women reported receiving a second dose of SP. The two predominant reasons given by women for not taking the second dose of SP were that they were not offered it (42%, 22/52) and that they were not informed a second dose was necessary (37%; 19/52) (data not shown). It is also possible that women who initiate ANC late account for some of this drop in effectiveness, as shown in Figure 2; indeed timing of the first ANC visit was a predictor of receipt of two doses of SP in the univariate analysis. The exclusion of administering SP in the 9^th^ month of pregnancy results in a narrow window of opportunity to administer SP to women who initiate ANC late. In the parallel study among health workers, there was considerable misinterpretation of the guidelines, that the 8^th^ month was inclusive i.e. that SP could not be given in the 8^th^ or 9^th^ month, and that the first dose should be denied unless a women could make two scheduled visits for both doses before the end of her pregnancy [32].

There were no statistically significant predictors of receiving two doses of SP in multivariate models, though both number (≥2 visits) and knowledge of malaria were associated with receipt in the univariate analysis, and late first ANC visit with non-receipt of SP. Predictors of receiving SP by DOT were marital status and distance from the health facility. Women who were married were less likely to receive either the first or second dose of SP by DOT, as were women who lived <5 km from their local health facility less likely to receive two doses of SP by DOT. Some studies have reported that women need their husband’s consent before taking drugs [37], [38], and it may be that unmarried women are more likely to take SP by DOT as they do not need to wait for their husband’s consent. Women who live close to a health facility may argue that they need to return home either to eat first, since women not wishing to take SP on an empty stomach was a problem observed by health providers in the parallel health facility study in Mali [32], or to ask their husbands consent to take the drugs. Interestingly, less poor women were less likely to receive SP by DOT in the health facility study undertaken at the same time of the household survey [33].

ITN use among pregnant and recently pregnant women in this setting was high, with over 90% of women who had attended ANC at least once reporting having used an ITN. The effectiveness analysis suggests that ANC is an effective channel for delivering ITNs to pregnant women, with 85% of pregnant and recently pregnant ITN users reporting having used an ITN from ANC. This is substantially higher use than reported in the 2006 DHS survey of 30% in Segou Region, as would be anticipated given ongoing scale up of the national ITN programme, and coverage was higher in rural areas compared to urban areas nationally (31% vs. 22%) [19]. This is consistent with our finding that ITN use was lower among recently pregnant women living within 5 km of a health facility, which usually have a central location in a village or town. Married, multigravid women were more likely to use an ITN in the univariate analysis, and adolescents less likely to use ITNs, suggesting that adolescents should be a high risk group for targeting ITNs in this setting. This is consistent with a recent systematic review and meta-analysis which showed using data from 19 countries of sub-Saharan Africa that married, multigravid, older women aged >19 years were the most likely to use an ITN [34]. The only multivariate predictor in our analysis from Segou District was household person: LLIN ratio which concurs with findings from another study using standardized national surveys across 15 countries, which showed that intra-household access to ITNs was the strongest and most consistent determinant of use among children [39].

A limitation of household surveys is potential reporting bias among interviewees, who may over- or under-report access and usage of interventions for a number of reasons including poor recall; this may be a general limitation but one which might be anticipated with regard to gestational age, which is difficult to interpret in the first trimester and especially among primigravidae. We measured concordance between reported and documented information using ANC cards where available, obtained from both pregnant and recently pregnant women. We found good concordance between reported and documented data for timing of first ANC visit (Kappa score 0.6945) and receipt of two doses of SP (K 0.682), and fair concordance for mean gestational age for the first and second dose of SP (K 0.249 and 0.206 respectively) [40]. There was potential selection bias among recently pregnant women; by selecting women with a child aged under one year, women who had had a pregnancy loss (miscarriage or stillbirth) may have been excluded from the recently pregnant group of women, and these women may have had different health seeking behaviours. Also, the diminishing numbers in the evaluative sample in the effectiveness analyses mean the regression analyses have less statistical power than anticipated. We use women’s self-report to interpret health provider practices which may not reflect true events, hence findings are compared with those of the observation studies in health facilities in the parallel study in Mali, which support the main findings from our analysis [32], [33].

## Conclusions

In conclusion, our analysis has shown that ANC is a more effective delivery channel for ITNs than for IPTp in this setting. This translates into a loss of community effectiveness of the IPTp strategy; with the current levels of coverage only 9% of the potential effectiveness of this strategy is being achieved, and a further 228 (95%) cases of LBW per 10,000 live births can be averted by achieving universal coverage of IPTp and ITNs. Loss in effectiveness is even greater if women who do not attend ANC were to be considered in this analysis. The practice among women of attending ANC early, an important element of focussed antenatal care, combined with evidence of health providers administering IPTp in the first trimester together with poor adherence to giving IPTp by DOT, points to the need to improve the quality of IPTp provision.

## References

[1] World Health Organisation (2004) A strategic framework for malaria prevention and control during pregnancy in the Africa region. Brazzaville: WHO Regional Office for Africa.

[2] Menendez C, D’Alessandro U, ter Kuile FO (2007) Reducing the burden of malaria in pregnancy by preventive strategies. Lancet Infect Dis 7: 126–135. 17251083 10.1016/S1473-3099(07)70024-5

[3] ter Kuile FO, van Eijk AM, Filler SJ (2007) Effect of sulfadoxine-pyrimethamine resistance on the efficacy of intermittent preventive therapy for malaria control during pregnancy: a systematic review. JAMA 297: 2603–2616. 17579229 10.1001/jama.297.23.2603

[4] Gamble C, Ekwaru PJ, Garner P, ter Kuile FO (2007) Insecticide-treated nets for the prevention of malaria in pregnancy: a systematic review of randomised controlled trials. PLoS Med 4: e107. 17388668 10.1371/journal.pmed.0040107 PMC1831739

[5] Worrall E, Morel C, Yeung S, Borghi J, Webster J, et al (2007) The economics of malaria in pregnancy–a review of the evidence and research priorities. Lancet Infect Dis 7: 156–168. 17251086 10.1016/S1473-3099(07)70027-0

[6] van Eijk AM, Hill J, Larsen DA, Webster J, Steketee RW, et al (2013) Coverage of intermittent preventive treatment and insecticide-treated nets for the control of malaria during pregnancy in sub-Saharan Africa: a synthesis and meta-analysis of national survey data, 2009–11. Lancet Infect Dis 13: 1029–1042. 24054085 10.1016/S1473-3099(13)70199-3

[7] Roll Back Malaria Partnership Refined/Updated Global Malaria Action Plan Objectives, Targets, Milestones and Priorities beyond 2011. Geneva, Switzerland, 2011.

[8] Countdown to 2015 (2010) Tracking progress in maternal, newborn and child health. 2010 report. Available: http://www.countdown2015mnch.org/reports-publications/2010-report/2010-report-downloads. Accessed 2012 May 2.

[9] Hill J, Dellicour S, Bruce J, Ouma P, Smedley J, et al (2013) Effectiveness of antenatal clinics to deliver intermittent preventive treatment and insecticide treated nets for the control of malaria in pregnancy in kenya. PLoS One 8: e64913. 23798997 10.1371/journal.pone.0064913 PMC3683044

[10] Sangare LR, Stergachis A, Brentlinger PE, Richardson BA, Staedke SG, et al (2010) Determinants of use of intermittent preventive treatment of malaria in pregnancy: Jinja, Uganda. PLoS One 5: e15066. 21124732 10.1371/journal.pone.0015066 PMC2993958

[11] Nganda RY, Drakeley C, Reyburn H, Marchant T (2004) Knowledge of malaria influences the use of insecticide treated nets but not intermittent presumptive treatment by pregnant women in Tanzania. Malar J 3: 42. 15541178 10.1186/1475-2875-3-42 PMC535531

[12] Tagbor H, Bruce J, Agbo M, Greenwood B, Chandramohan D (2010) Intermittent screening and treatment versus intermittent preventive treatment of malaria in pregnancy: a randomised controlled non-inferiority trial. PLoS One 5: e14425. 21203389 10.1371/journal.pone.0014425 PMC3010999

[13] Chico RM, Chandramohan D (2011) Intermittent preventive treatment of malaria in pregnancy: at the crossroads of public health policy. Trop Med Int Health. 10.1111/j.1365-3156.2011.02765.x 21477099

[14] Smith Paintain L, Antwi GD, Jones C, Amoako E, Adjei RO, et al (2011) Intermittent Screening and Treatment versus Intermittent Preventive Treatment of Malaria in Pregnancy: Provider Knowledge and Acceptability. PLoS One 6: e24035. 21887367 10.1371/journal.pone.0024035 PMC3161113

[15] Smith LA, Jones C, Adjei RO, Antwi GD, Afrah NA, et al (2010) Intermittent screening and treatment versus intermittent preventive treatment of malaria in pregnancy: user acceptability. Malar J 9: 18. 20074372 10.1186/1475-2875-9-18 PMC2817700

[16] Gross K, Alba S, Schellenberg J, Kessy F, Mayumana I, et al (2011) The combined effect of determinants on coverage of intermittent preventive treatment of malaria during pregnancy in the Kilombero Valley, Tanzania. Malar J 10: 140. 21599999 10.1186/1475-2875-10-140 PMC3126755

[17] Marchant T, Schellenberg D, Nathan R, Armstrong-Schellenberg J, Mponda H, et al (2010) Assessment of a national voucher scheme to deliver insecticide-treated mosquito nets to pregnant women. CMAJ 182: 152–156. 20064944 10.1503/cmaj.090268 PMC2817322

[18] Webster J, Kweku M, Dedzo M, Tinkorang K, Bruce J, et al (2010) Evaluating delivery systems: complex evaluations and plausibility inference. Am J Trop Med Hyg 82: 672–677. 20348517 10.4269/ajtmh.2010.09-0473 PMC2844570

[19] Cellule de Planification et de Statistique, Ministère de la Santé, Direction Nationale de la Statistique et de l’Informatique, Ministère de l’Économie dlIedC, Bamako Mali. (2007) Enquête Démographique et de Santé du Mali 2006.

[20] Ridde V, Girard JE (2004) [Twelve years after the Bamako initiative: facts and political implications for equity in health services accessibility for indigent Africans]. Sante Publique 16: 37–51. 15185584 10.3917/spub.041.0037

[21] Programme National de Lutte Contre le Paludisme, MInistere de al Sante, Organisation Mondiale de la Sante (2006) Polititique Nationale de Lutte Contre le Paludisme au Mali.

[22] Baiden F, Owusu-Agyei S, Bawah J, Bruce J, Tivura M, et al (2011) An evaluation of the clinical assessments of under-five febrile children presenting to primary health facilities in rural Ghana. PLoS One 6: e28944. 22174932 10.1371/journal.pone.0028944 PMC3236777

[23] Filmer D, Pritchett LH (2001) Estimating wealth effects without expenditure data–or tears: an application to educational enrollments in states of India. Demography 38: 115–132. 11227840 10.1353/dem.2001.0003

[24] Vyas S, Kumaranayake L (2006) Constructing socio-economic status indices: how to use principal components analysis. Health Policy Plan 21: 459–468. 17030551 10.1093/heapol/czl029

[25] World Health Organisation (2006) Malaria in Pregnancy: Guidelines for measuring key monitoring and evaluation indicators.

[26] Roll Back Malaria, MEASURE Evaluation, USAID UNICEF, World Health Organization, et al. (2009) Guidelines for Core Population-Based Indicators. RBM/WG/2009/TP.1.

[27] Gikandi PW, Noor AM, Gitonga CW, Ajanga AA, Snow RW (2008) Access and barriers to measures targeted to prevent malaria in pregnancy in rural Kenya. Tropical Medicine & International Health 13: 208–217. 18304267 10.1111/j.1365-3156.2007.01992.x PMC2607535

[28] Guyatt HL, Noor AM, Ochola SA, Snow RW (2004) Use of intermittent presumptive treatment and insecticide treated bed nets by pregnant women in four Kenyan districts. Trop Med Int Health 9: 255–261. 15040563 10.1046/j.1365-3156.2003.01193.x

[29] Webster J (2011) A voucher scheme for insecticide treated nets in Ghana: development of a methodology for delivery systems evaluation PhD thesis, London School of Hygiene and Tropical Medicine.

[30] Chandramohan D, Webster J, Smith L, Owusu-Agyei S, Awine T, et al (2007) Is the Expanded Programme on Immunisation the most appropriate delivery system for intermittent preventive treatment of malaria in West Africa?. Tropical Medicine and International Health 12: 1–8. 17550471 10.1111/j.1365-3156.2007.01844.x

[31] Hill J, Kazembe P (2006) Reaching the Abuja target for intermittent preventive treatment of malaria in pregnancy in African women: a review of progress and operational challenges. Trop Med Int Health 11: 409–418. 16553924 10.1111/j.1365-3156.2006.01585.x

[32] Webster J, Kayentao K, Diarra S, Diawara SI, Haiballa AA, et al (2013) A qualitative health systems effectiveness analysis of the prevention of malaria in pregnancy with intermittent preventive treatment and insecticide treated nets in mali. PLoS One 8: e65437. 23843941 10.1371/journal.pone.0065437 PMC3701011

[33] Webster J, Kayentao K, Bruce J, Diawara SI, Abathina A, et al (2013) Prevention of malaria in pregnancy with intermittent preventive treatment and insecticide treated nets in mali: a quantitative health systems effectiveness analysis. PLoS One 8: e67520. 23840729 10.1371/journal.pone.0067520 PMC3695962

[34] Hill J, Hoyt J, van Eijk AM, D’Mello-Guyett L, Ter Kuile FO, et al (2013) Factors affecting the delivery, access, and use of interventions to prevent malaria in pregnancy in sub-Saharan Africa: a systematic review and meta-analysis. PLoS Med 10: e1001488. 23935459 10.1371/journal.pmed.1001488 PMC3720261

[35] Kayentao K, Garner P, van Eijk AM, Naidoo I, Roper C, et al (2013) Intermittent preventive therapy for malaria during pregnancy using 2 vs 3 or more doses of sulfadoxine-pyrimethamine and risk of low birth weight in Africa: systematic review and meta-analysis. JAMA 309: 594–604. 23403684 10.1001/jama.2012.216231 PMC4669677

[36] World Health Organisation (2012) Updated WHO Policy Recommendation (October 2012). Intermittent Preventive Treatment of malaria in pregnancy using Sulfadoxine-Pyrimethamine (IPTp-SP).

[37] Diala C, Pennas T, Choi P, Rogers S (2012) Barriers to uptake of malaria prevention and treatment during pregnancy in Cross River and Nasawara States, Nigeria.

[38] Iliyasu Z, Gajida AU, Galadanci HS, Abubakar IS, Baba AS, et al (2012) Adherence to intermittent preventive treatment for malaria in pregnancy in urban Kano, northern Nigeria. Pathogens and Global Health 106: 323–329. 23182135 10.1179/2047773212Y.0000000037 PMC4005129

[39] Eisele TP, Keating J, Littrell M, Larsen D, Macintyre K (2009) Assessment of insecticide-treated bednet use among children and pregnant women across 15 countries using standardized national surveys. Am J Trop Med Hyg 80: 209–214. 19190215

[40] Altman DG (1991) Relating change to initial value. Nephron 59: 522. 1758557 10.1159/000186630

